# Perceptions of artificial intelligence in healthcare: findings from a qualitative survey study among actors in France

**DOI:** 10.1186/s12967-019-02204-y

**Published:** 2020-01-09

**Authors:** M.-C. Laï, M. Brian, M.-F. Mamzer

**Affiliations:** 1 Cordeliers Research Centre, INSERM, Sorbonne University, USPC, University Paris Descartes, University Paris Diderot, ETRES Host Team, 75006 Paris, France; 2 Paris District Court, Paris, France

**Keywords:** Artificial intelligence, Grounded theory, Health professionals, Patients, Responsibility

## Abstract

**Background:**

Artificial intelligence (AI), with its seemingly limitless power, holds the promise to truly revolutionize patient healthcare. However, the discourse carried out in public does not always correlate with the actual impact. Thus, we aimed to obtain both an overview of how French health professionals perceive the arrival of AI in daily practice and the perception of the other actors involved in AI to have an overall understanding of this issue.

**Methods:**

Forty French stakeholders with diverse backgrounds were interviewed in Paris between October 2017 and June 2018 and their contributions analyzed using the grounded theory method (GTM).

**Results:**

The interviews showed that the various actors involved all see AI as a myth to be debunked. However, their views differed. French healthcare professionals, who are strategically placed in the adoption of AI tools, were focused on providing the best and safest care for their patients. Contrary to popular belief, they are not always seeing the use of these tools in their practice. For healthcare industrial partners, AI is a true breakthrough but legal difficulties to access individual health data could hamper its development. Institutional players are aware that they will have to play a significant role concerning the regulation of the use of these tools. From an external point of view, individuals without a conflict of interest have significant concerns about the sustainability of the balance between health, social justice, and freedom. Health researchers specialized in AI have a more pragmatic point of view and hope for a better transition from research to practice.

**Conclusion:**

Although some hyperbole has taken over the discourse on AI in healthcare, diverse opinions and points of view have emerged among French stakeholders. The development of AI tools in healthcare will be satisfactory for everyone only by initiating a collaborative effort between all those involved. It is thus time to also consider the opinion of patients and, together, address the remaining questions, such as that of responsibility.

## Introduction

Recently, extremely divergent ideas and points of view confront each other when the burning topic of AI is discussed. The most alarmist individuals, who denounce the advent of transhumanism, find themselves in disagreement with the most cautious, who explain that we overestimate the abilities of AI. As a consequence, many would like to define general principles for AI [1]. However, even defining what AI really means is not straightforward; the lack of a clear definition is indeed a first obstacle to overcome.

According to the definition of Marvin Minsky, the father of AI, AI simply means that a machine is able to do a task which is considered to be an intelligent one by human beings. Indeed, AI is a discipline for which the applications fall into two categories: (1) the attempt to reproduce the capabilities of the human mind and 2) the creation of tools to carry out tasks which today need a human action. AI has been divided into many sub-disciplines, focusing on very distinct problems (such as vision, problem solving, language comprehension, learning, etc.). There is no unified paradigm of research and some branches of AI have become places of multidisciplinary exchange where philosophers, psychologists, computer scientists, and others who are interested in the various issues of AI can meet [2]. AI can also be understood as a concept, i.e. a general and abstract idea that the human mind makes of a concrete or abstract object of thought that enables it to associate the various perceptions that it has of that object. It was during the Dartmouth Conference (in 1956) that John McCarthy and Marvin Minsky invented AI, not only as a discipline but also as a concept [3].

Science has recently witnessed striking advances in the ability of machines using AI to understand and manipulate data using algorithms. Many fields of activity stand to benefit immensely from deep learning, a branch of AI which uses neural networks and data. Artificial neural networks are flexible mathematical models that use multiple algorithms to identify complex nonlinear relationships within large datasets (analytics) [4]. Thus, with the increasing amount of data created every day by society, AI’s performance has grown using machine learning techniques. Today, AI is able to offer concrete and ingenious applications that have gradually become intertwined within our daily lives. We can cite the example of targeted ads on the Internet, the proposition of films and series which should please us by Netflix according to what we have seen before, the identification of credit-card fraud on the Internet, etc. These applications have already proven their efficiency in various areas, leading to growing fascination among the public. Thus, many countries, such as the United States and China, have invested rapidly in these techniques [5, 6].

AI seems to have already rapidly inserted itself everywhere into patient healthcare, starting a few years ago. It could be argued that this may just be the result of a mere passing fad. However, it appears that the will to develop its application within the healthcare system is still very strong [4, 7–9]. For example, the journal Nature published an article in 2017 in which machine learning (an AI technique) was able to diagnose skin cancer as efficiently as dermatologists [10]. In 2018, another scientific article claimed that AI was even able to do it better than dermatologists [11]. In addition, the FDA (Food and Drug Administration) in the USA authorized the first AI device to diagnose diabetic retinopathy without a physician’s help in April 2018 [12].

At the same time, numerous companies in France have shown their interest in the applications of AI in healthcare. Consequently, according to key opinion leaders, some specialties could be totally replaced by AI devices, leading to a professional upheaval for the affected physicians, with the most concerned specialties being radiology and pathology [13]. Thus, in France, some of these specialists are doing everything they can to be prepared for this change, as in many other countries [14, 15].

Although the United States is at the forefront, France is now trying to catch up, turning to the immense amount of health data gathered by the administration and public services in France, the National System of Health Data [16]. The amount of data within this system keeps increasing and it already includes the data from reimbursed healthcare (including health insurance data), the medical cause of death (CépiDC), disability data from the Independent-Living Support Fund (Caisse Nationale de Solidarité pour l’Autonomie—CNSA), and a sample of data from supplementary health insurance organizations. In addition, after the report of Cédric Villani [17], the French President, E. Macron, announced the creation of a “Health Data Hub” as a strong point of the French global AI strategy. It will be a trusted third party between health data producers, actors wishing to use such data, and citizens or civil-service representatives. The first mission of the “Hub” will be to promote the gathering of clinical data, which are the data collected during the course of care, “in a completely anonymous manner” [18, 19].

At the same time, new issues arise from the application of AI in healthcare, the main ones being difficulties with the use of health data (*e.g.* data liability, privacy concerns) [20], concerns about cybersecurity, the question of responsibility, and the integration of AI tools into current practice and ethics considerations [21, 22]. For example, Google recently published a list of ethical principles related to the development of AI in June 2018 [23].

Currently, AI tools and the related above-mentioned issues are still within the realm of research and representation, but it is commonly accepted that these tools will revolutionize medical practice and the medical community is beginning to take this potential seriously [24]. However, medicine has not been integrating the tools as quickly as the technology has been advancing. In addition, without the involvement and cooperation of health professionals, AI will never be integrated into current practice. Similarly, the legislative and regulatory frameworks will also have to be included and the public interest bodies concerned involved in this general discussion.

Thus, our primary objective was to provide an overview of how health professionals perceive the arrival of AI in their practices and what influences their views. Indeed, their perception may condition their future adoption of AI tools, which in turn will lead to a revolution in medical practice [30]. These professionals are, in a sense, the gatekeepers who can decide what is good for their practice and thus their patients.

The secondary objective of this study was to define possible barriers from the perspective of the various stakeholders, as governments begin to promote AI, by identifying points of convergence and divergence between them.

No similar study has yet been conducted in France or abroad, and it is essential to take these aspects, too often ignored, into account. Indeed, despite a rapidly increasing number of scientific publications related to this topic, none have focused on and compared the various interests and points of view of the main actors about the role of AI in medicine and healthcare. This is the first qualitative research which gathers and cross-references these different points of view [7, 25–29].

## Methods

We sought to determine the lie of the land by collecting testimonials in a broad manner to obtain an inventory of the issues of concern to stakeholders. The goal was to obtain a better understanding of the obstacles for the development of AI in healthcare. Such an understanding could allow more rational investment in AI. Thus, we needed to assemble various points of view. The grounded theory method (GTM) appeared to be best suited for this study because, with this method, the theory emerges from the fieldwork [31].

The study was conducted in Paris (France). First, people of interest were identified based on a demonstration of their knowledge and strong interest in AI through several public symposia about AI or data and health in France. Most were directly involved in AI in industry or as researchers. They were contacted by email to schedule an appointment to discuss the subject of AI in healthcare. Those that did not answer the first time were sent a reminder. The interviews took place mainly face-to-face at the participants’ workplace or, if this was not possible, they were conducted by telephone. Indeed, stakeholders were interviewed by telephone if they were outside Paris at the time of the interview or if they were reluctant to be interviewed face-to-face, mainly because of their schedule. Semi-directive interviews were then conducted using an interview guide built from a bibliographic search so that major themes would be discussed, such as changes in practice, data security, etc. After a few introductory remarks, the interviews were conducted according to the guide. The questions were open-ended, allowing the various stakeholders to develop their ideas, or even to digress and venture into subjects not always directly related to the initial question. Memos and field notes were also used during the interviews. The participants were free to express themselves in a discussion. Depending on their role as a stakeholder, some aspects were more developed than others (cf. Box, which shows an example of the questions we asked).

Second, if a topic had not appeared to have been sufficiently covered during an interview or the discussion had raised new questions requiring further investigation, we sought new stakeholders by Internet to explore further the issue; they were chosen based on their experience in the issues raised by these specific questions and were supposed to be concerned by AI because of their training or their function. Thus, physicians, institutional representatives and other individuals were interviewed in the second round. Radiology was the most prevalent specialty among the interviewed physicians; radiology is indeed one of the medical specialties most involved in AI.

The first data were collected by voice recordings, with the agreement of the participants, and the corresponding interviews re-transcribed. Then, the transcribed interviews were analyzed using the GTM, as revised by C. Lejeune [32], respecting the anonymity of the participants.

The data were analyzed using three stages of the coding process, according to the methodology described in various studies [33–35]: initial (or open) coding, intermediate (or axial) coding, and advanced (or selective) coding. In the initial coding stage, raw data were produced and labels (codes) attached to them. In the intermediate coding stage, significant codes were chosen and assembled to form categories. In the advanced coding stage, categories were developed from the codes and a core category was selected. Finally, a theory was developed that established the links between the categories. The interviews were no longer conducted when the “categories” which had emerged were “saturated”, meaning that the interviews did not introduce anything new.

Box. Frame for the question grid

1. What is AI?
2. What is human intelligence? What are the unique features of human intelligence?
3. Is there a need to set limits on the place of AI place in medicine? What impact can physicians have on these changes?
4. Is the profession of imaging physicians changing?
5. Is further training required for physicians?
6. On what criteria can we assess such tools?
7. What is the potential impact of AI on the physician–patient relationship?
8. Should AI be anticipated in a context in which it is not yet in place? What is the point of anticipating and not waiting for legislation, as we often do?
9. Health Data and Privacy: Is there reason to be so suspicious?
10. Concerning the challenges of increasingly personalized medicine (excluding curative), how far can we go in prevention?
11. Do you think of other ethical issues?
12. Are there any other changes that would be necessary to prepare for the arrival of AI?
13. What are the challenges in terms of responsibilities that AI could give rise to (for the doctor, designer, industry, etc.)?

## Results

Forty people were interviewed between September 2017 and June 2018, until saturation was observed. They consisted of: 13 physicians—5 radiologists, 1 anatomopathologist, 2 surgeons, 1 dermatologist, 1 oncologist and radiotherapist, 1 nuclear physician, 1 geriatrician, 1 cadiologist—seven individuals involved in industry—1 consultant specialized in AI, 1 vice-president in charge of the AI branch, 1 company founder in AI, and 4 employees in a company providing medical imaging solutions using AI—five researchers of AI in health; seven members of regulatory agencies (institutional representatives) related, or not, to health (CNIL, ANSM, Asip santé, HAS, ministry of health), and eight people who were not directly involved (without any conflict of interest according to the definition of Bion et al. [36]) and had thought or written about the topics of AI and data. Among them were: 2 ethicists, 1 retired public health physician, 2 public health researchers working on confidentiality issues, 1 representative of patients, 1 lawyer, 1 researcher in automation in aeronautics working on the comparative advantages of using AI. Twenty-four interviews were conducted face-to-face and 16 by telephone. The interviews lasted between 30 and 70 min.

As expected, the “perception of AI by health professionals” emerged as a core category on which all others depended. These main ideas found in the various identified categories are presented and classified in Table 1, showing the priorities, driving forces, points of vigilance, and obstacles identified by them.

**Table 1:** Summary of the stakeholders’ interests by category

Stakeholders’ common and specific points of view about AI					
Stakeholders	Health professionals/Physicians	Health industry	Individuals without conflict of interest	Regulatory agencies	Health researchers
General					
Common notions for all stakeholders	Fuzzy notion of AI—Issues around health data—International competition—Development of AI—Change in healthcare relationship—Radiology as a precursor				
Specific					
Priorities	Give the best care to patients	Be efficient	Protect individuals and their rights, but also improve health	*Regulate appropriately* (legislating or providing guidelines)	Generate research results
Driving forces	Will to integrate these tools into practice	General will to develop AI	None	Omnipresence of the subjects (some actions have already been led)	Presence of encouraging results (due to big data and machine learning)
	Possibility of a change in medical training	State of the art of AI in healthcare		Development of AI’s applications	Existence of active work on AI
Points of vigilance	Waiting for proof in current practice	*None*	Promote population’s education/information	Not to succumb to the ambient willingness to legislate	Not to call everything “AI”
			Promote population’s informed opinion		
			Respect privacy		
			Evaluate issues of social justice		
Obstacles (scientific and/or legal)	*Questions of liability/responsibility*	*Questions of liability/responsibility*	*Questions of liability/responsibility*	*Not feeling able to evaluate AI’s software yet*	Financial context: need of funding
				Misreading of institutional support	
		*Regulatory context*		Difficulty to entirely understand the subject	
		Difficulty to access to annotated health data (need for professionals)			

*Related to the Law*—Source: [25]

*The following results exclusively represent the views of the interviewees. The opinions of the authors have been relegated to the Discussion.*

1. Most shared ideas developed during the interviews focused on the myth surrounding AI, the need to find a balance between access to data and their protection, and the potential interference with the physician–patient relationship.

For most of the individuals interviewed, the perception of AI benefits, in part, from its representation in popular culture. There appears to a collective fascination with AI. Indeed, the frequently denied myth [37] is still alive and with it the hope to achieve the perfect human being. Indeed, as many of the interviewees stated, it is very important to debunk this myth in people’s minds to allow them to have a clearer insight about what it is. Most were speaking about “AI” as if they were speaking of an independent entity rather than a set of various technological applications (tools), thus contributing to the perpetuation of the myth. In addition, the interviews highlighted the fuzzy notion of AI. For example, there was confusion between “weak AI”^1^ and “strong AI”.^2^

Despite such confusion and inaccuracy, all participants agreed that an immense amount of individual health data were essential to develop reliable AI tools for health. However, they also pointed out that a balance still needs to be found between widening access to data and ensuring confidentiality and respect for privacy.

Moreover, most of the people interviewed (physicians, industrial partners, participants without a conflict of interest, and researchers) expressed the opinion that AI development is increasing because of international competition. AI tools could have an impact on the organization of the healthcare system, as they are not intended to be developed only in the care setting. Thus, for them, the public could well take advantage of these tools (*e.g.* self-screening). AI could also interfere with the physician–patient relationship. In the case of machine learning, for example, the “black box” phenomenon could prevent the doctor from providing clear information to his patient, depending on the degree of the tool’s independence in the final result. These expected developments could thereafter cause various ethical problems, depending on the type of information provided by the AI tool. At the time of this study, all the interviewees agreed that radiologists would likely be the first to work with those new tools and thus be confronted with these issues.

2. Healthcare professionals don’t deny the promise of AI, but they mostly care about providing the best care for their patients and highlight the gap between public declarations and current practice.

Physicians appeared to have a positive view about what AI tools could bring to patients. For many healthcare professionals surveyed, AI tools developed by industrialists would be able to save time for the doctor, carry out watchful and alert work, better monitor the population, alleviate some deficiencies related to medical deserts, and even improve management difficulties in the healthcare sector (especially at the hospital). AI could therefore be a means to enter an era of more effective medicine, improving care and reducing costs, while increasing patient safety. For some physicians, AI would therefore represent a revolution in their practices and patient care, whereas for the others, it would only be a continuation of the ongoing improvements in medical practice. Nevertheless, all possibilities of working with AI tools envisaged during the interviews were based on ongoing research. Healthcare professionals then pointed out that few of these projects have yet proven to be successful in real life and it was clear to the interviewees that AI is still in its infancy. Moreover, physicians mentioned that there is a discordance between scientific advances and the thundering announcements made in the media. The buzz, particularly generated by some companies, does not correspond to the reality of operational technological advances, which diverge highly from what is currently experienced in hospitals. Yet, to date, no healthcare professional appears to be able to visualize what the AI of tomorrow would really change in his/her practice, and most of the ideas put forward by health professionals were close to the current societal discourse. Because giving the best care to their patients emerged in the interviews as the primary goal of physicians, they were not opposed to change and were often ready to reconsider their role, as long as it remains central. They believe that AI should not become a “consumer good” that health professionals would not need. During the interviews, some radiologists complained about the fact that, too often in radiology, there is a tendency to focus on the innovative aspect of a tool and not its utility (what does it bring to the patient?). For the healthcare professionals concerned (in particular, radiologists), the primary interest of physicians in AI is not just a pointless desire but comes from the fact that, today, the ability of physicians to establish diagnoses is made complicated by the massive flow of data. Thus, as they said, they need tools to analyze and classify such data. Moreover, some physicians believe that they should first ask themselves what the needs and possible changes are, rather than go directly to the AI tools. Thus, if AI tools become crucial in medical decisions, physicians stated that they were not prepared (would not agree?) to be held criminally responsible if a medical error was made by an AI tool. Thus, if physicians were obliged to use AI tools, they are very open to training to better understand how they work. They also believe that society will only accept mistakes from a machine if it understands why such mistakes may occur. For example, there are master’s degrees in the field to help future doctors understand how AI tools work.

Finally, physicians remained cautious about the message being delivered by those in industry who, depending on the context, may or may not have suggested that some AI tools will replace physicians (*e.g.* medical imaging).

3. For healthcare industrial partners, AI is a true breakthrough and the real challenge is access to health data.

From the point of view of those interviewed who are in the health industry, AI is going to revolutionize medical practice and be a true breakthrough thanks to the progress of research in this field. According to them, the real challenge they face is access to health data for the purpose of training machine-learning algorithms. Indeed, those in industry need health data, and this is the main difficulty for them. Above all, however, when it comes to “supervised” machine-learning techniques, they need data labelled by physicians to obtain results that are as precise as possible (*e.g.* diagnosis, proposed treatment, etc.). Another pitfall that was always brought up was the sense of excessive regulation concerning health data used by private companies in France. However, at the same time, they had the impression of there being a legal loophole and a lack of clarity of the legal documents. They considered that current laws are able to address the new issues that arise with AI, thus triggering a will to legislate in the hope of devising a better framework for AI in France. Paradoxically, they said that the question concerning responsibility in case of injury was not yet relevant. For them, AI tools are only meant to help doctors with their decisions and not to replace them. The rule of law should therefore remain unchanged in their view. In addition, those in industry were quite clear about their not being ready to be held responsible for their AI tools if such a tool induced harm to a patient because of an unpredictable evolution of the tool due to a “black box” phenomenon. They also pointed out that their being considered to be partially responsible in case of injury would hinder the development of health AI tools in France.

Talking about the future role of physicians, the position of the industrial partners was not always clear, depending on the medical specialty. Concerning medical imaging in particular, it was clear to them that AI tools could replace radiologists, but that such replacement will not happen for a long time because society is not yet ready to accept this type of medical care. Indeed, and unlike physicians, those in industry did not appear to see why it would be meaningful for a physician to understand how a new AI tool works. Sometimes during the interviews, they mentioned mere superficial learning. For example, they believed that radiologists were mainly “here to push a button”.

4. Participants without a conflict of interest highlight the imprecision of the notions and the need for education and have major concerns about the role of AI in health, social justice, and freedom.

All the participants without a conflict of interest admitted they were influenced by the discussions surrounding the subject. Thus, society appears to have certain preconceived notions, the most widespread being that “more automatic” was equivalent to “more secure”, which is questionable. Some interviewees said that it was necessary to avoid rejecting AI out of hand. They felt there was a need to build an operational definition to help people understand what AI can really do for patients. Thus, the education of the public has to be considered upstream, for example concerning the requirements of data (Which data? What standard of quality? etc.), as well as the need to step back and understand that AI does not possess the absolute truth.

They pointed out the question of data ownership as being very difficult, because people believe that their data belong to them, whereas this is legally not true. The notion of “non-belonging health data” appeared to be problematic. Indeed, some participants highlighted that this notion did not respond to social reality. Indeed, the common belief is that an individual owns his/her health data. Moreover, clarification was also needed about the duty to inform or give consent following the application of the GDPR.^3^

Another matter of concern that the population should be aware of is the aspect of social justice. Some participants agreed that the primary motivation for the development of AI was financial. Thus, given the intrinsic logic of AI, namely the prioritization of the collective above the individual, they questioned whether there will remain a place in society for individual vulnerabilities.

It is thus necessary to think upstream about the balance between private life and health gains to define what would be acceptable or not for society. An individual should then be able to make his/her own choice because there is also a question of freedom that arises with this subject. In addition, other questions were raised concerning patients: would their choice really be considered, knowing it may be biased by the fact he/she is ill? Will the government make a choice concerning an individual when it comes to public health choices, instead of the individual himself/herself? It is therefore the responsibility of the regulatory authorities to also protect individuals, and the one of physicians to keep valuing the individual in order to allow patients to make their own choices.

From the point of view of the patient association representative, it was also necessary that patient associations, authorities, and industrial partners agree on what AI really means. Indeed, it is very difficult for patients to follow the debates and express an informed view on the subject as long as industry does not adopt a more responsible posture when talking about AI and promise what they cannot deliver to patients (the advantages without the disadvantages). Moreover, patients felt that they were not sufficiently consulted by industry, especially concerning the evolution of these tools.

5. Members of regulatory agencies are beginning to take an interest in the subject but appear to be currently overwhelmed.

The primary role of the regulatory agencies will be to provide recommendations and regulate the implementation of AI. However, one of the interviewees shortened the interview by saying that AI was pure speculation and that it has not been a topical issue thus far. AI was still a relatively unclear concept for several other interviewees. The posture they adopted could be defined as more-or-less informed expectation. It appeared that some work groups were emerging, but in the absence of concrete integration of AI into care, the regulatory agencies appeared to have a relatively poor grasp of the subject. However, some participants reported that there were already actions underway to facilitate the development of AI, even though they were not always visible to those in industry, who complained of a lack of proactivity from the ministries. Although regulatory agencies were not particularly in favor of succumbing to the ambient willingness to legislate, some participants suggested that regulatory agencies could rely more on soft law, as well as guidelines, to be more helpful and visible for both healthcare industrialists and physicians.

Nonetheless, some of members of the regulatory agencies expressed that they will likely be the first to be involved in the assessment of AI tools concerned by the “black box” phenomenon. However, if healthcare professionals are to use these tools, the regulatory agencies know that it will be necessary for them to be able to trust the assessment process, as in the past. This appeared to be a fundamental point for integrating AI into the current practice of healthcare professionals and health regulatory agencies sometimes appeared to be ill prepared to take on this huge responsibility.

6. Researchers in AI have a pragmatic vision of what AI is and are focused on their own research.

A major reproach of health researchers in AI was that the kind of AI media were talking about had nothing to do with the kind of AI they were working on, which has a much more specific and narrow definition. They appeared to be confident about the progress in their research, regretting the too-slow translation from research to practice, even if working with healthcare industry was seen as a way to accelerate the translation. They also complained about the difficulties induced by legislation when it comes to collecting data for researches. According to them, this was the only way to see their research funding increase and thus allow effective development of AI and a guarantee of its quality. They did not participate in the discussion about general concerns and focused on their research approach.

Only one person, a researcher working in the aeronautics field, spoke about the human–computer interaction (HCI). For him, even though HCI is not specific to AI, questioning the automation of tasks and limits should be considered as one of the main goals of the integration of AI into healthcare tools. He explained that, in this discipline, one of the main ideas is to delegate only when necessary. If not, there is a risk of deskilling. Deskilling is the loss of competence of the human who does not know how to carry out a task that he did before because he stopped performing it for the benefit of the machine. Thus, the research said that before integrating AI into medical practice, it would be important to ask ourselves what can be transferred, to identify repetitive tasks that AI can do without risking the loss of skills by the physicians. Therefore, the physician interviews often highlighted the need to have AI tools which would fit in with practice, like any other tool, so that healthcare professionals could use them.

## Discussion

Healthcare applications with embedded AI are currently being developed worldwide and bring with them a number of professional, societal, and ethical questions. Some countries are more advanced in this domain than others [6] and France is not considered to be a pioneer in the “AI for health” landscape. Nevertheless, France is on the verge of entering into international competition. Thus, interviewing French stakeholders involved in this topic was much more than an exercise of moral philosophy, as they are enlightened by both their own knowledge of the topic and feedback from abroad.

We used GTM, which is a systematic methodology that operates inductively. It has the unique feature of not leaving aside any opinion and of not relying on the number of times an idea appears. This study was conducted in accordance with GTM, as revised by C. Lejeune, which refutes the usefulness of the triangulation of both methods and sources^4^ if the objective is not to arrive at an absolute truth [32]. Our aim was to draw up a “state of play”, a type of inventory, and render the points of view exchanged during these interviews with the best possible accuracy. It is both different from and complementary to theoretical studies because it proves the interest of working together, not theoretically, but using a bottom-up approach. In 2016, an English study proved that “studying ethics empirically “from the ground,” within the ethical landscape provides more plural and differentiated pictures”. This 2016 study concluded:”if […] policymakers want to make defensible decisions they need to make them whilst also being responsive to and ideally in conversation with other actual agents” [38]. Thus, because we present the point of view of the involved actors, institutions and others concerned, it is possible to grasp the subject in its numerous dimensions and address the expressed ethical concerns that a top-down approach would never show.

Our results show that all the stakeholders interviewed share many concerns, regardless of their role within the healthcare system: the fuzzy notion of AI, issues concerning health data, the knowledge that AI is developing, especially in radiology, the reality of international competition, and the upheaval in the doctor-patient relationship. Moreover, despite the diversity of stakeholders interviewed, and although the French system has its own unique features, most of our results are consistent with those of the international literature. Thus, the main shared idea was that AI tools are expected to change the landscape of diagnosis and decision making for both physicians and patients and to affect all stakeholders in the healthcare field [7, 25, 26, 30]. Nonetheless, aside from this obvious convergence of opinions, there were many divergences among the various categories of stakeholders concerning their priorities, driving forces, points of vigilance, and obstacles, corresponding to their specific and professional interests and responsibilities (*cf*. Table 1).

### Specific and professional interests of the various stakeholders and their interactions

#### Industrial partners and researchers are driving the development of AI tools for health

This study is the first to gather opinions from these different perspectives, providing important insight into the point of view of the various stakeholders. Based on the Results, Table 2 identifies the sources of motivation and pressure for the various stakeholders in the development of AI. It reveals a strong interdependence between these stakeholders because of their common willingness to develop AI tools for health while staying true to their values. Industrial partners are clearly driving the development of AI in healthcare, through interactions with all the other interviewed stakeholders. They play a key role in the rapid growth of AI and appear to be committed to developing AI tools to meet physicians’ needs. Their commitment to AI is sometimes considered by other stakeholders as a source of motivation and sometimes as a source of pressure. At the same time, the industrial partners need the physicians’ cooperation, not only to access reliable data, but also to integrate these tools into current practice. They also need regulatory agencies to create a more permissive regulatory framework. Finally, although industrial partners are very proactive, they gauge their level of investment with their level of responsibility. Indeed, if they are held responsible, they fear it would slow down their initiatives in the field of AI. Similarly, researchers appear to be highly motivated and optimistic about the capacities of AI. According to them, the two essential factors shaping the development of AI for health are access to data and the development of successful algorithms. Thus, they campaign for accessing health data and, together with industry, their commitment to AI is very strong and can be a source of pressure for the others. Once again, these results are consistent with those of previous studies, as some US scientists recently predicted a three-level impact for these tools: “for clinicians, predominantly via rapid, accurate image interpretation; for health systems, by improving workflow and the potential for reducing medical errors; and for patients, by enabling them to process their own data to promote health” [27].

**Table 2:** Relationships between the stakeholders concerning their sources of motivation and pressure to develop AI

	Physicians	Industrial partners	Individuals without conflict of interest	Members of regulatory agencies	Health researchers
Sources of motivation	Patients’ needs	Health researchers	Patients	Physicians	Industrial partners
	Industrial partners		Regulatory agencies	Industrial partners	
Sources of pressure	Industrial partners	Physicians	Industrial partners	Industrial partners	Regulatory agencies
		Regulatory agencies			

#### Among physicians, radiologists appear to be the least reluctant to integrate AI tools into practice

In this study, the most prevalent specialty among interviewed physicians was radiology, which is the most advanced specialty in terms of AI tool development. They are not opposed to integrating AI tools into practice and to promoting a change in medical training to make the adoption of those tools more natural. However, for physicians, AI tools have to be useful. These results are consistent with previously published statements of radiologists. Indeed, these health professionals have been the first to be exposed to the AI revolution and they already agree that AI could be a useful assistant; this positive attitude is perceptible both in France [14] and abroad [28]. The general practitioners’ (GPs) view of AI may be more skeptical, as suggested by a UK study in which GPs claimed they would only expect AI to improve the efficiency of their work and reduce the administrative burden [29].

#### Members of regulatory agencies feel that they will have a significant role to play in the management of the healthcare system

Members of regulatory agencies appear to be aware of their responsibilities in the process of evaluating AI software. Their position concerning the question of lawmaking is consistent with that of the US Food and Drug Administration (FDA), which does not establish new requirements or any official policy regarding AI in healthcare, but rather has taken the first step toward developing a draft guidance [39]. It attempts to develop “an appropriate framework that allows the software to evolve in ways to improve its performance, while ensuring that changes meet the [FDA’s] gold standard for safety and effectiveness throughout the product’s lifecycle” [40, 41]. Moreover, certain AI tools could alter the organization of the healthcare system in France. Indeed, in France, physicians are still the gatekeeper of the healthcare system by law, whereas it has begun to change abroad, especially with direct-to-consumer health AI tools. Thus, in England, the Babylon Diagnostic and Triage System AI tools have started to slip out of the doctors’ hands to be used directly by individuals, although GPs consider the performance of these tools in terms of clinical evaluation is not yet sufficient to ensure confidence in patient safety [42] [43]. Therefore, the question of regulation must be considered to avoid chaotic evolution of the healthcare system. French regulatory agencies show a strong willingness to not be pinned down by the development of AI, especially as they admit they have difficulties to entirely grasp the subject of AI in healthcare. Currently, the interviewees may misread the level of institutional support, some actors complaining about their lack of proactivity and their so-called obstructive attitude. Moreover, laws are a means of protecting the individuals from excesses. People are looking for guarantees of protection since they are not completely at ease with the fact that an intelligent tool could decide when healthcare is involved [44]. Thus, laws are a way to provide people with a guarantee that their values will be respected, especially when it comes to a complex subject such as AI in healthcare.

### A strong need to define the responsibilities of each stakeholder

Unsurprisingly, responsibility appeared as a core question, and four of the five categories of stakeholders (physicians, industrial partners, institutions, and individuals without a conflict of interest) highlighted it as a potential obstacle to the development of AI in the field of health. Clearly, none wanted to be held responsible in the event of an injury due to the use of an AI tool. Indeed, physicians are ready to take the blame in case of injury, but only if they can understand the choices made by the AI tool. For those in industry, this core concern of who should be held responsible in case of an error caused by an AI-driven medical device could even hinder the development of AI. This issue of accountability, and more largely of responsibility, resonates in many countries, including China [25, 45–47], and also concerns the members of French regulatory agencies who clearly want to be able to evaluate AI software before going forward. In the US, from the physician’s perspective, this issue could even reduce the level of medical innovation if “the “safest” way to use medical AI from a liability perspective is as a confirmatory tool to support existing decision-making processes, rather than as a source of ways to improve care” [48]. For individuals without a conflict of interest, the main concern is to protect the population, for example, by creating a victim compensation fund if necessary. Opinion pieces published in various countries also concern this burning topic [49]. Legal protection of individuals may become necessary as healthcare professionals cannot entirely fulfil the role of protector.

### Ethical values are driving physicians and individuals without conflict of interest

This study is the only to gather and study the values and perceptions of various actors concerning AI tools for healthcare. It will help us to define the values and principles to respect to facilitate the adoption of AI. Responsibility was a value shared by many stakeholders, but it is not the only one that emerged from this study. Similarly, the question of the values driving the various stakeholders have equally been addressed in the French and international medical literature [14, 22, 50]. French physicians have an ethical imperative as they first want to provide the best possible care to patients. Despite their enthusiasm for AI, they remained pragmatic, as they specified that they were still waiting for proof of efficiency in current medical practice. This result is aligned with those of an online cross-sectional survey conducted in the UK about point-of-care tests, in which many GPs declared that they were reluctant to use available point-of-care tests (for diagnosis, reducing referrals, monitoring, management) in their current practice because of, among other things, the potentially misleading results and the limited usefulness of the tools [51]. Individuals without conflict of interests also have an ethical imperative to protect individuals while keeping them in good health. They have a unique position among stakeholders. Unlike the others, they have no direct involvement and so speak with more detachment. They were deemed by the other stakeholders to be neither a source of motivation nor a source of pressure (c*f*. Table 2). They represent one of the most concerned groups of stakeholders, although they usually do not participate in the development of AI (lawyers, patient representatives, ethicists, etc.) and thus their opinion is poorly known. In our study, they did not mention any drivers for AI development. This is probably because they see themselves more as a “watchdog”, and thus they have a strong desire to protect individuals from potential risks driven by AI. The same tendency can be observed in several international scientific publications: individuals without a conflict of interest more actively focus on areas of tension rather than on drivers [49, 52]. In our study, French individuals without a conflict of interest were the actors who provided the largest number of ideas concerning points of vigilance. They believe the population should be educated to be able to express an informed view on the subject. In the guidelines proposed by the European Union to favor trustworthy AI, the focus on education is even wider: they propose to educate all stakeholders and raise awareness “to foster an ethical mind-set”. This means educating not only those involved in making the products but also the users and other affected groups [53]. French individuals without a conflict of interest also mentioned another point of vigilance. They believe the population will be more in favor of AI tool development if it preserves their private life. For them, the entry into force of the GDPR on May 25, 2018 is not a sufficient guarantee of such protection at a time when governments encourage data sharing without being able to entirely protect these data. Nonetheless, they have ideas about how AI should be developed to protect individuals and, most of all, they are able to speak about AI in healthcare for everyone. This highlights the interest of involving a third party in collective decisions. This is consistent with the principles of democracy in public health.

### To develop AI, it will be necessary for people to collaborate using a translational approach

It is regrettable that no patient’s opinion was collected in this study, but at the time of the interviews, they had not yet formulated an opinion on the subject and did not want to express themselves, despite attempts to collect their opinion. We also regret the absence of GPs among the physicians interviewed. A unique feature of this study is that it gathered the opinion of industrial partners in France, which has not been done before. Even if it is difficult to know the opinion of those in industry abroad, all the companies concerned in this study had an international scope. Similarly, this study collected the opinions of both individuals without a conflict of interest and regulatory agencies. This study was conducted in 2017 and 2018, a period not always covered in recent articles, and thus provides information about the latest evolution of the topic [54]. It allows us to compare our results to what is currently occurring in other countries, making it not just an “exercise in experimental moral philosophy”. It throws a spotlight on what we could gain by these actors working together in France. This is not the first time that this conclusion has emerged. The same analysis appeared in theoretical studies carried out in other countries, such as China, Australia, and England [25, 43, 55]. For example, an opinion paper published in 2019 concluded that “we must bring together diverse expertise, including workers and citizens, to develop a framework that health systems can use to anticipate and address issues” [43]. In our study, French physicians mentioned the need to integrate AI tools into practice. Similarly, a study in China stated that what they call “the productization process of AI in medicine” requires its “integration into complex existing clinical workflows” [25]. Nonetheless, neither study, nor those interviewed, elaborated on this aspect, although it appears to be considered essential. The only person who could talk about integrating AI tools in the medical workflow in our study was not part of the healthcare system. He was a researcher working on human–computer interaction in aeronautics, but he could provide some information about the subject. Human–computer interaction is not specific to AI; it is a discipline that examines the automation of tasks and the limits which should be imposed in this area. Indeed, aeronautics is particularly aware of the difficulties related to AI and the automation of systems. Thus, this work shows the added value of translational research and the need to use a transdisciplinary approach. The definition of translational research appears to be an ever-evolving phenomenon. “Nowadays it is defined by a process that starts with fundamental research (genes, molecular processes, biochemical pathways) and ends at a macro level (social healthcare, access to healthcare, access to education, and so on)” [56]. We must therefore be able to work together and interact with other disciplines to foster a translational approach to develop AI.

## Conclusion

AI tools are reaching the medical field. It is now a reality that we must face to facilitate their arrival. While a certain level of hyperbole seems to have taken over the discussion of AI in healthcare, we also found that diverse considerations and knowledge have emerged among each category of stakeholder. On the one hand healthcare industrials and researchers highlight the need for high-quality health data; on the other hand, physicians are still waiting for evidence of the usefulness of these tools, and wonder if they will be held responsible in case of an injury due to an AI tool that they do not fully understand. Members of regulatory agencies would like to be able to play their role as regulators both in the development of AI tools and in the race towards health data. Individuals without conflict of interest wonder how collective and individual interests will be balanced as the development of AI for the benefit of patients will result in part from this balance.

Combining big data and AI in healthcare could lead to an important breakthrough for both patients and professionals. However, although we identified many of the driving forces for the development of AI in healthcare, the above-mentioned obstacles could also hinder it, especially if the values of the stakeholders are not respected. AI and big data must be integrated and used in an ethical manner if we want to develop AI tools that are going to be satisfactory for all actors. Thus, in the coming years, society will have to be vigilant concerning the place given to big data and AI. Since AI continues to move forward, it is up to all actors involved to define the essential points for a fair form of healthcare consistent with their values as we identified in this work. Thus, we will have to “cross the valley of death” [57] to ensure that everyone communicates and collaborates in a way that avoids missing any essential points. Ethical considerations will play a significant role by helping us circumvent potential obstacles in the adoption of AI tools in healthcare. Thereby, the remaining questions that still tears them apart, such as question of the sharing of responsibilities, will have to be addressed. We will have to join a conversation among all stakeholders concerned, without exception. Thus, it seems all the more important to focus on patient voices. Their opinions on AI in healthcare were difficult to gather at the time this study was conducted and are still largely unknown. Further work will therefore have to address their expectations so that the development of AI is for the benefit of patients and not in spite of them.

## Data Availability

The datasets generated and analysed during the current study are not publicly available due to their nature (individual records) but are available from the corresponding author on reasonable request.

## Abbreviations

AI: artificial intelligence
GTM: grounded theory method

## Footnotes

1 “Weak AI” is AI without self-awareness that focuses on a specific task.

2 “Strong AI” is self-conscious and its machine's intellectual capacity would be functionally equal to that of a human.

3 The EU General Data Protection regulation. For more information, go to https://eugdpr.org/.

4 Triangulation is a procedure for the validity of knowledge produced by research. Triangulation of methods involves using more than one methodological approach to analyze “data”. Triangulation of sources means that data are collected from several different sources.
